# How Does Income Inequality Influence Environmental Regulation in the Context of Corruption? A Panel Threshold Analysis Based on Chinese Provincial Data

**DOI:** 10.3390/ijerph18158050

**Published:** 2021-07-29

**Authors:** Shi Wang, Wen Zhang, Hua Wang, Jue Wang, Mu-Jun Jiang

**Affiliations:** 1 School of Economics and Finance, Xi’an International Studies University, Xi’an 710128, China; wangshi1107@xisu.edu.cn (S.W.); zhangwen01@stu.xisu.edu.cn (W.Z.); 2 Institute of Communication and Global Public Opinion, Xi’an International Studies University, Xi’an 710128, China; 3 “One Belt One Road” Economic and Trade Cooperation Innovation Team, Xi’an International Studies University, Xi’an 710128, China; 4 School of Foreign Studies, Xi’an Jiaotong University, Xi’an 710049, China; roy1998228@sina.com; 5 International Business School, Shaanxi Normal University, Xi’an 710119, China

**Keywords:** income inequality, environmental regulation, corruption, threshold effects

## Abstract

The question of how the income inequality of residents affects the level of environmental regulation in the context of official corruption was the core research issue of this study. We analyzed this problem using the panel threshold regression model from 26 provinces in China from 1995 to 2017. We found that when there is no official corruption, the widening of the residents’ income inequality promotes stricter environmental regulations; when the corruption problem is serious, the expansion of the residents’ income inequality leads to the decline in environmental standards; that is, the impact of residents’ income inequality on environmental regulation has a threshold effect due to corruption. In addition, the threshold effect due to corruption of all residents’ income inequality on environmental regulation is mainly generated by the urban residents’ income inequality and the urban–rural income inequality. This paper contributes to the literature that concentrates on the relationship between income inequality and environmental regulation, and shows that corruption is a key factor that can deeply influence that relationship. The research conclusion shows that increasing anti-corruption efforts can not only maintain national political stability, social fairness, and justice, but also be a powerful measure for environmental pollution governance.

## 1. Introduction

The achievements associated with China’s economic development since its reform and opening up are world-renowned, and the material living standards for the majority of the population have been greatly improved. However, at the same time, environmental pollution caused by economic development has become more serious. The past five editions of the Environmental Performance Index (EPI), published by Yale University every two years, showed that China’s environmental quality ranked 105th in all counted countries in 2008 (105/149), 121st in 2010 (121/163), 116th in 2012 (116/132), 118th in 2014 (118/178), 109th in 2016 (109/180), 120th in 2018 (120/180), and 120th in 2020 (120/180). It can be observed that although China’s relative ranking has increased since 2014, it is still in a backward position among all countries. In particular, air pollution needs to be mentioned. In the 2018 report, China’s air quality was ranked fourth from the bottom of all of the counted countries. Long-term exposure to polluted air has made residents north of the Huai River more susceptible to various cardiopulmonary diseases, and life expectancy has therefore decreased by 5.5 years [1]. In addition, according to the 2019 China Eco-Environmental Status Bulletin, issued by the Ministry of Ecology and Environment of China, in 2019, 53.4% of China’s 337 prefecture-level cities exceeded the air quality standard, causing 2118 city × days of severe and above pollution. Air pollution has caused a significant increase in the incidence of respiratory diseases. In addition, the suspension of classes at primary and secondary schools and kindergartens in many places, and the temporary closure of factories and the restriction of vehicles in cities, have seriously affected the health and normal life of the people.

In addition to environmental pollution, China also has a large income inequality between residents, which has become another important factor affecting the push for sustainable economic development and social stability. According to data released by the National Bureau of Statistics of China, the Gini coefficient of all residents in China has been higher than the international warning level of 0.4 since 2000, and it has approached 0.5 in some years. The results estimated by economists are even more serious. The China Household Finance Survey (CHFS) of the Southwestern University of Finance and Economics revealed that the disposable income of the top 10% of all households accounted for 57% of all households’ disposable income in 2017.

The dual pressures of environmental pollution and income inequality raise the question of whether there is some internal connection between income inequality and environmental pollution. Most studies believe that income inequality will weaken environmental regulations and increase environmental pollution [2,3,4]. They believe that environmental quality is one of the results of the interaction between residents’ income distribution and market forces. The income inequality affects the environmental consumption preferences of all income classes, leading to destruction of the environment. Specifically, the low-income class aims to improve their material living standard to narrow the income inequality with the wealthy class at the expense of the environment, and the high-income class also accumulates wealth by destroying the environment and transfers assets to countries or regions of high environmental quality. In addition, the cost of environmental pollution is mainly borne by the low-income class, but the benefits are mainly attributed to the high-income class. Because the latter have greater political influence, environmental protection is often ignored when formulating economic policies, resulting in greater environmental pollution. However, some scholars believe that income inequality helps reduce pollution emissions. They find that high-income residents often have a stronger awareness of environmental protection, so the widening in income inequality increases the emission reduction efforts of the entire society [5].

In general, controversy exists about how income inequality affects environmental quality. In addition, the impact of income inequality on the environment may vary due to differences in political systems and economic development. This means that there may be a threshold effect on the impact of income inequality on the environment: when a certain factor is at different levels, the impact of income inequality on the environment is different. Eriksson and Persson [6] and You et al. [7] show that the impact of income inequality on environmental pollution depends on the level of democracy. However, their research is applicable to cross-country studies rather than studies within a country, because in a country such as China, there is no significant difference in the level of democracy between different provinces. Instead, in the current study we used the alternative indicator of official corruption to examine the impact of income inequality on environmental regulation in the context of corruption, and to unify the contrasting findings of the existing literature. The main contribution of this paper is therefore to quantify the corruption threshold effect of residents’ income inequality on environmental regulation in a developing country such as China. The study’s conclusion can be used as a reference for other similar developing countries. In addition, we also identify the main source of the corruption threshold effect.

The remainder of this paper is structured as follows. Section 2 reviews the research literature in this field and provides a brief theoretical analysis of the corruption threshold effect of residents’ income inequality on environmental regulation. Section 3 describes the specification of the econometric model, variables, data sources, regression results, and analysis of empirical analysis in detail. Finally, Section 4 concludes and proposes possible policy recommendations.

## 2. Literature Review and Theoretical Analysis

### 2.1. Literature Review

This paper is related to three branches of literature. The first is the research about the impact of income inequality on environmental pollution and environmental regulation. The former, i.e., the research about the impact of income inequality on environmental pollution, can be further divided into three types of views. The first is that income inequality increases environmental pollution. The groundbreaking theoretical research by Boyce [2] found that income inequality increases pollution emissions through two mechanisms: one is that income inequality affects people’s environmental demand functions. At a given average income level of residents, the expansion of income inequality means that the rich become richer and the poor are economically worse off than they were before. This reduces the environmental needs of the poor and tends to result in the overuse of resources and the environment. Although the environmental needs of the rich are rising, they are more willing to directly transfer assets to areas of higher environmental quality, rather than investing to improve the environmental quality of the region. The other mechanism is that, because the cost of deteriorating environmental quality is mainly borne by the poor, the benefits are mainly attributed to the rich. Compared with the poor, the rich often have more political resources and social influence. For their own best interests, they will hinder the formulation and implementation of environmental protection policies, which further deteriorates the quality of the environment. The results of empirical analysis implemented by Torras and Boyce [3] supported the above theoretical research conclusions and found that narrowing the income inequality is conducive to improving the environment. Vona and Patriarca [4] constructed a dynamic model to study the relationship between income inequality and environmental technology innovation, and found that there is a non-linear relationship between the two factors, and the higher income inequality of residents is not conducive to environmental technology development. Eriksson and Persson [8] constructed a median voter model and found that the democratization reform which brings low-income group into the franchise can improve environmental performance only when the marginal utility elasticity of consumption is small. Based on the data of the US, Baek and Gweisah [9] found that a more equitable distribution of income reduces harmful emissions in the short and long term. Based on China’s panel data from 1995 to 2010, Zhang and Zhao [10] found that income inequality increased China’s carbon dioxide emissions, and income inequality in the eastern region had a greater impact on carbon dioxide emissions than in the western region. Based on the data of the BRIC countries from 1980 to 2014 and the quantile-on-quantile regression technique, Mallick et al. [11] found that an increase in income causes a decline in environmental quality. Using China’s panel data from 1996 to 2014, Liu et al. [12] also found that the increase in income inequality leads to a deterioration in environmental quality. Using quantile regression over the 1996–2014 period, Ekeocha [13] found that inequality engenders more environmental degradation across all quantiles in Africa. Cheng et al. [14] investigated the impact of income inequality on both direct and indirect CO_2_ emissions of 30 provinces in China from 2000 to 2015 using the STIRPAT (Stochastic Impacts by Regression on Population, Affluence, and Technology) model and the panel quantile regression method. They found that income inequality significantly promotes direct CO_2_ emissions under all quantile levels, and the impact on indirect CO_2_ emissions is not significant. Zhang and Zhang [15] found that there is a long-run positive relationship between income inequality and carbon emissions in China.

The second view is that income inequality helps reduce environmental pollution. Environmental quality is seen as a public rather than a private product, so the argument that the income inequality affects the individual’s environmental demand function is untenable, and thus there is no other class that can avoid the consequences of environmental degradation through other means. High-income residents often have a stronger environmental awareness, so the increase in income inequality will not worsen the environmental quality, but may reduce pollution emissions [5]. In Hubler’s [16] research, the pooled regressions support the negative inequality–emissions nexus. Ravallion et al. [17] believed that income inequality enables high-income residents and low-income residents to simultaneously reduce pollution emissions, which is beneficial to the improvement of environmental quality. Based on panel data from 92 countries between 1991 and 2015, Huang and Duan [18] found that there is a significant negative correlation between income inequality and carbon emissions. Furthermore, there is a nonlinear threshold effect in their relationship.

The third view is that the impact of income inequality on environmental pollution is uncertain, and the specific direction depends on other factors. Eriksson and Persson [6] found that the impact of income inequality on environmental pollution depends on the degree of democracy. When the degree of democracy is low, income inequality contributes to the improvement of environmental quality, whereas when the degree of democracy is high, income inequality exacerbates the deterioration of environmental quality. Based on the balanced data and the spatial panel regression model of 31 provinces in China from 1996 to 2015, Liu et al. [19] found that there is an inverted U-shaped relationship between income distribution and environmental quality, which indicates an appropriate level of income inequality helps to improve environmental quality. Studies have also shown that income inequality has different effects on the pollutant emissions from different countries [20]. Liu et al. [21] used panel ARDL and quantile regression models to analyze the impact of income inequality on carbon emissions in the US. They found that rising income inequality increases carbon emissions in the short term and decreases them in the long term. Using panel data and spatial panel models covering 41 B&R (Belt and Road Initiative) countries, You et al. [7] found that the level of democracy affects the nonlinear relationship between income inequality and CO_2_ emissions. More precisely, the interaction of high inequality and poor democratic institutions increases pollution emissions. It should be noted that this study is not contradictory with the study by Eriksson and Persson [6] mentioned above: Eriksson and Persson [6] analyzed whether income inequality has a different impact on environmental performance in countries with different degrees of democracy. You et al. [7] studied the moderating effect of democracy on the inequality–emissions nexus. Nonetheless, both of these studies inspired the current study. Using a panel smooth transition regression, Rojas-Vallejos and Lastuka [22] found that income inequality in low- and middle-income countries reduces per capita carbon emissions, whereas in high-income countries, it promotes carbon emissions. In recent studies, Grunewald et al. [23], Uddin et al. [24], Baležentis et al. [25], and Imran et al. [26] also found a non-linear relationship between income inequality and emissions, although different data and research methods were applied.

The latter, i.e., the research about the impact of income inequality on environmental regulation, is relatively rare. To our knowledge, only He et al. [27] has analyzed this issue. By constructing a theoretical model of public choice, they found that when the level of corruption is low, the rise of income inequality strengthens environmental regulation. When the level of corruption is high, the impact of income inequality on environmental regulation is uncertain. The empirical analysis based on cross-country data validated the conclusion of the theoretical analysis.

The second branch is the study about the influence of corruption on income inequality. This can be divided into two views. The first view is that corruption increases the income inequality, which is supported by most scholars. Tanzi [28] believed that corruption-related gains were often attributed to bribed officials and business owners, both of whom are located in a high-income strata, so corruption increases the income inequality. Glaeser and Saks [29] found that corruption was an important reason for the expansion of income inequality in the United States. Blackburn and Forgues-Puccio [30] constructed a dynamic theoretical model to analyze the impact of corruption on income distribution, and found that the high-income group avoided all kinds of taxes that should be paid through bribery, and the reduction of government revenue reduced the utility of its income redistribution function. Both mechanisms widen the income inequality. Batabyal and Chowdhury [31] conducted empirical analysis with panel data of 30 countries from 1995 to 2008, and found that corruption indirectly increases income inequality by inhibiting financial development. Using panel data covering the period 1996–2018 and MENA (Middle Eastern and Northern Africa) countries, Lassoued [32] found that a lower level of corruption is associated with less income inequality.

Another view holds that the relationship between corruption and the income gap is not a simple linear relationship, but an inverted U-shaped relationship. For example, Chong and Calderón [33] and Li et al. [34] found an inverted U-shaped relationship between corruption and income inequality based on cross-country data. The reason for this is that in countries with low levels of corruption, people concentrate on formal production activities. Therefore, the economy reaches a high level of equilibrium and income distribution is fair. In countries with a high level of corruption, people concentrate on various rent-seeking activities. As a result, the economy reaches a low level of equilibrium and income distribution is also relatively fair. The income inequality of countries with a moderate level of corruption is higher than that of the former two.

The third branch is the study of the impact of corruption on environmental regulation. Fredriksson and Svensson [35] constructed a three-stage game model to investigate the impact of political stability and official corruption on environmental pollution. The subsequent empirical analysis using cross-sectional data from 63 developed and developing countries showed that the increase in corruption significantly reduces the intensity of environmental regulation. However, when the political instability increases, the expected profits of polluters decline, leading to the weakening of the incentive to bribe corrupt officials, thus weakening the impact of environmental regulation. Pellegrini and Gerlagh [36] used panel data from 22 European countries to conduct an empirical study and found that corruption had a negative impact on environmental regulation, and was an important source of differences in environmental regulation among European countries. The empirical study of Oliva [37] also found that corrupt activities reduced the effectiveness of control policies of automobile exhaust emissions. Using city-level data for the period from 2009 to 2016 in China, Zhou et al. [38] found that the anti-corruption campaign has reduced air pollution by 20.3%, partially through the increased intensity of environmental regulations. Liu and Dong [39] obtained similar results based on a geographically temporally weighted regression model.

This paper contributes to the existing literature in the following three aspects: First, in terms of research ideas, the literature review above shows that the existing literature mainly analyzes the impact of income inequality on environmental pollution, and there is almost no literature which concentrates on the impact of income inequality on environmental regulation (with the exception of He et al. [27]). Although the public is more concerned about environmental quality, from the government’s perspective, environmental regulation is more specific and controllable. Therefore, this study was devoted to analyzing the impact of income inequality on environmental regulation, which is more practically significant. Furthermore, this study considered how corruption affects the impact of income inequality on environmental regulation. Second, this study took 26 provinces in China from 1995 to 2017 as the research sample, to examine the threshold effect of income inequality on environmental regulation from the perspective of domestic regions. This approach can resolve the heteroscedasticities that may exist in the cross-country research conducted by He et al. [27]. Finally, an increasing amount of literature has used modern econometric methods to scientifically study environmental pollution [40,41,42]. Specifically, the panel threshold regression model proposed by Hansen [43] was adopted in this study, and various control variables, such as the economic development level, industrial structure, population density, unemployment rate, and foreign direct investment, were incorporated into the model. This allowed a more accurate investigation of the threshold effect of income inequality on environmental regulation.

### 2.2. Theoretical Analysis

Overall, previous scholars have already carried out a large amount of research on the environmental impact of residents’ income disparity, but they have obtained two completely opposing views. Although Eriksson and Persson [6] and You et al. [7] unified the two aspects of the studies, the conclusion is that the impact of income inequality on environmental pollution depends on the degree of democracy, which is obviously not applicable to different regions in the same country. However, it provides ideas for further research; that is, the impact of residents’ income inequality on environmental pollution may have a threshold effect, such that the environmental impact of residents’ income inequality changes with the change in a threshold variable. Because the degree of democracy is a national-level variable, research at the regional level cannot be used. Nonetheless, the degree of democracy essentially affects pollution emissions by weakening the environmental regulation. At the regional level, the level of official corruption is a key variable that affects environmental policies and environmental standards. Corruption of the central government leads to the stranding of various environmental policies, delay in the issuance of policies, or less forceful policies than expected. Corruption of the local government affects the specific implementation of environmental policies in enterprises. When the level of corruption is high, high-pollution enterprises that were previously unable to produce can organize production. Enterprises that originally used cleaner production technology are able to stop using emission-reduction equipment or switch to more polluting production technologies to save costs, which in turn leads to increased pollution emissions. Therefore, this study used the corruption level of officials in various regions as a substitute, to explore the threshold effect of corruption on the impact of residents’ income inequality on environmental pollution.

When there is comparatively little corruption at the local level, environmental regulations can reflect everyone’s environmental preferences. According to He et al. [27], the environmental standard is determined by the intermediate voter at this time. When the inequality of income distribution is high, the income of intermediate voters is low. To reduce the negative effects caused by environmental pollution, the stricter the environmental regulations expected by the intermediate voters, the higher the environmental standards. On the contrary, if environmental regulations are determined by officials who may accept bribes, the higher the inequality of income distribution, the more money the wealthy class, particularly the polluting business owners, use to bribe government officials to weaken environmental regulations, and the lower the environmental standards.

Based on the above analysis, the following hypothesis can be proposed: when the level of official corruption is low, the higher the income inequality, and the stricter the environmental regulations. When the official corruption is serious, the higher the income inequality, and the lower the environmental standards.

## 3. Empirical Analysis

### 3.1. Specifications of the Econometric Model

To empirically study the relationship between residents’ income inequality and environmental regulations, this study used the following measurement equation:

(1) $$ER_{it}=\beta_{0}+\beta_{1}Gini_{it}+\beta_{2}COR_{it}+\beta_{3}Z_{it}+u_{i}+\varepsilon_{it}$$

where the subscripts *i* and *t* denote provinces and time, respectively. The dependent variable *ER* is environmental regulation. *Gini* is the Gini coefficient of residents’ income. *COR* is the official corruption level. *Z* is another variable that affects environmental regulation, detailed in Section 3.2. $u_{i}$ is a fixed effect of the region that does not change with time, and $\varepsilon_{it}$ is a random disturbance term.

The hypothesis of the theoretical analysis shows that the impact of residents’ income inequality on environmental regulation depends on the level of official corruption, rather than being linear. When the level of official corruption is low, the expansion of the income inequality of residents is conducive to the improvement of environmental standards. When corruption is more serious, the expansion of residents’ income inequality weakens environmental regulation; that is, the impact of residents’ income inequality on environmental regulation has a threshold effect due to corruption. To empirically verify this threshold effect, this study used the panel data threshold regression model proposed by Hansen [43] to address the corruption threshold based on the data’s own structure, and then analyze the relationship between different income inequality levels and environmental regulations. It should be noted that, in previous research, the specific threshold value was subjectively determined by the researcher, and parameter estimation and hypothesis testing were not performed on the threshold value. Obviously, the threshold value obtained by this method is not scientific or reasonable. The panel data threshold regression model proposed by Hansen [43] is based on the structure of the data itself to obtain the threshold value, which makes the determination of the threshold value more scientific and reasonable, and provide regression results that are more credible. However, this model requires that explanatory variables must be exogenous variables, which is difficult to satisfy in practice. Taking a single threshold as an example, the panel threshold regression model can be expressed as:

(2) $$\{\begin{array}{c} y_{it}=\rho_{i}+{\hat{\tau }}_{1}x_{it}+\omega_{it},\gamma_{it}\leq \mu \\ y_{it}=\rho_{i}+{\hat{\tau }}_{2}x_{it}+\omega_{it},\gamma_{it}>\mu \end{array}$$

where $\gamma_{it}$ is the threshold independent variable and μ is the threshold value to be estimated. The model requires that the explanatory variable is an exogenous variable; that is, $x_{it}$ is not related to $\omega_{it}$ in Equation (2). By combining the regression Equation (1), a single threshold regression model is obtained:

(3) $$\{\begin{array}{c} ER_{it}=\beta_{0}+\beta_{11}Gini_{it}+\beta_{2}COR_{it}+\beta_{3}Z_{it}+u_{i}+\varepsilon_{it},COR\leq \mu \\ ER_{it}=\beta_{0}+\beta_{12}Gini_{it}+\beta_{2}COR_{it}+\beta_{3}Z_{it}+u_{i}+\varepsilon_{it},COR>\mu \end{array}$$

The threshold independent variable is *COR*. μ is the threshold value of corruption to be estimated. According to the hypothesis of the theoretical analysis, the expectation of $\beta_{11}$ is positive and that of $\beta_{12}$ is negative. The multiple threshold regression model can be extended based on the above formula.

It should be noted that recent studies on income inequality and environmental pollution, such as those of You et al. [7] and Liu et al. [19], are based on the spatial econometric model. The advantage of the spatial econometric model is that it can analyze the spatial spillover effect of environmental pollution or environmental regulation. In the case of environmental pollution, the environmental quality of one region must be closely related to the environmental quality of the neighboring regions due to the influence of natural factors. Regarding environmental regulation, under China’s power structure and performance evaluation mechanism, local governments may compete to lower environmental standards to attract foreign direct investment. This may lead to the “race to the bottom” of environmental regulation; that is, the weakening of environmental regulation in one region may lead to the weakening of environmental regulation in neighboring regions. However, the aim of this paper is to study the threshold effect of income inequality on environmental regulation. The panel threshold regression model proposed by Hansen [43] has been widely adopted in the existing literature to analyze the threshold effect [44,45]. In addition, due to the limitations of the current econometric analysis techniques, we were not able to incorporate the spatial spillover effect into the panel threshold regression model. Therefore, this study only used the panel threshold regression model for analysis.

### 3.2. Variable Description

(1) Environmental regulations. During the examined study period, China’s environmental regulations were mainly command-and-control and market-based regulations [46,47,48,49]. The former refers to environmental laws enacted by government departments and is also the most widely used environmental regulation in China. The latter consists mainly of two policies, namely, emissions fees and the emissions trading scheme. Considering the availability of data and the applicability of indicators, referring to Xie et al. [44] and Feng and Chen [50], this study used the proportion of industrial pollution investment in industrial added value (ER1) of each province as a measure of command-and-control environmental regulation, and the proportion of sewage charge income in industrial added value (ER2) as a measure of market-based environmental regulation. The latter was also used to test the robustness of the empirical results.

(2) Resident income inequality. There are some studies that use microdata from the Chinese Household Income Project survey (CHIPs), which is conducted by China’s National Bureau of Statistics, to calculate income inequality in China [51,52,53]. However, the CHIPs only released data in 1988, 1995, 2002, 2007, and 2013. In addition, the CHIPs only released data for some of the 31 provinces in mainland China. For example, the CHIPs only covered 16 provinces in 2007 and 2013. As a result, this microdata is not applicable to this study. Globally, the most commonly used measurement of the income distribution gap of residents is the Gini coefficient, which indicates the degree of deviation between the average income inequality of different groups and the overall average income. The specific calculation process is to first divide all residents into N groups and calculate $x_{i}$, which is the average income of each group. Referring to Liu et al. [12] and Liu et al. [19], the formula for calculating the Gini coefficient of residents’ income is:

(4) $$Gini=\frac{1}{2\lambda N^{2}}\sum_{i=1}^{N}\sum_{j=1}^{N}\left|x_{i}-x_{j}\right|$$

where λ is the average of all income. The grouping in the above formula is obtained by the equal division method, but in reality, the data often appears in the form of non-equal grouping. Referring to Zhang and Zhao [10] and Thomas et al. [54], the corresponding calculation formula is:

(5) $$Gini=\frac{1}{\lambda }\sum_{i=2}^{N}\sum_{j=1}^{i-1}Q_{i}\left|x_{i}-x_{j}\right|Q_{j}$$

In the above formula, $Q_{i}$ indicates the proportion of the population of group *i*.

Due to the inconsistency in the means of grouping urban residents’ income and rural residents’ income in statistical yearbooks in different periods in China, neither the equal grouping calculation of Equation (4), nor the non-equal grouping calculation of Equation (5), can provide a unified result. Therefore, we used the following formula in the calculation process:

(6) $$Gini=1-\frac{1}{QY}\sum_{i=1}^{N}(Y_{i-1}+Y_{i})Q_{i}$$

where *Q* is the total population, *Y* is the gross national income, and $Y_{i}$ is the income accumulated to group *i*. For the calculation of the Gini coefficient, Equation (6) needs only to be grouped according to residents’ income, and the number of people in each group and the average income must be known.

It should be noted that the income of urban and rural residents is separately counted in the China Statistical Yearbook. Therefore, using Equation (6) can only be used to separately calculate the Gini coefficients of urban and rural residents of each province. In addition, we also need to use the group weighting formula proposed by Sundrum [55] to calculate the Gini coefficient:

(7) $$G=Q_{U}^{2}\frac{\delta_{U}}{\delta }GU+Q_{V}^{2}\frac{\delta_{V}}{\delta }GV+Q_{U}Q_{V}\frac{\delta_{U}-\delta_{V}}{\delta }$$

where *G* represents the Gini coefficient of income of all residents, *GU* and *GV* represent the Gini coefficient of income of urban and rural residents respectively, $Q_{U}$ and $Q_{V}$ represent the proportion of urban and rural population, respectively, and $\delta_{U}$, $\delta_{V}$, and δ represent the per capita income of urban residents, rural residents, and all residents in a certain area, respectively.

In the specific calculation process, this study first used the income distribution data of urban residents and rural residents from a sample survey of the statistical yearbooks of provinces and cities. Equation (6) was used to directly calculate the Gini coefficient of the income of urban and rural residents in each province, and Equation (7) was then used to calculate the Gini coefficient of all residents’ income. Due to the availability of data, this paper obtained the Gini coefficient values of 26 provinces from 1995 to 2017.

Research by Sicular et al. [56] showed that the urban–rural income inequality in China is an important cause of the large income inequality of all residents, so it is necessary to include the urban–rural income inequality indicators of various provinces. This study used the ratio of the per capita disposable income of urban residents to the per capita net income of rural residents (UV) as the measurement variable of the urban–rural income inequality, which can also be used to test the robustness of the empirical results for Gini coefficient.

(3) Official corruption. In China, official corruption is often highly concealed, and it is difficult to accurately estimate how much corruption exists in various regions. To measure the level of corruption in Chinese provinces, an objective indicator that can measure corruption is needed. There are three common methods of measuring corruption: judicial data, social evaluation, and corruption indexes. Because the prosecution of corruption crimes by judicial authorities depends on many factors, judicial data has rarely been used in the established literature. Social evaluation may be influenced by personal experience, rumors, or the indirect effects resulting from the observation of corruption, so it is difficult to objectively reflect the status of corruption. Corruption indexes are generally obtained through questionnaires, field surveys, interviews, or data analysis, so they are relatively fair and are often used in empirical analysis among countries. Among these corruption indexes, the more well-known corruption indicators are the Corruption Perception Index (CPI) proposed by Transparency International (TI), the Control of Corruption Index proposed by the World Bank, the International Country Risk Guide (ICRG) index issued by PBS, the Corruption Index in the World Competitiveness Yearbook published annually by the Institute for Management Development (IMD) in Lausanne, Switzerland, and the Corruption Index in the Global Competitiveness Report published by the World Economic Forum. Among these indexes, the CPI index and the ICRG index are the most widely used.

Although the CPI index and ICRG index are relatively objective and fair, these indexes only disclose the overall corruption situation of each country (or region) each year, and are not detailed to the regional level within a country. Therefore, they are mainly used in country-level empirical analysis. Because the research in this paper requires corruption data of officials at the provincial level, these indexes cannot be used. Cai et al. [57] used the proportion of entertainment and travel costs in the sales of firms, which was taken from firm-level surveys conducted by the World Bank during 2000 to 2005, to measure the level of corruption in Chinese firms. The disadvantage of this data is that it can only be used to measure the level of corruption in enterprises, rather than the level of corruption in provinces. In addition, the survey was only conducted in a portion of Chinese cities and was not conducted after 2005. Therefore, the data was not applicable to the current study. Fisman and Gatti [58] used the number of public officials who abused power in each state when measuring the level of corruption in the United States. The current study drew on their method. The work reports of each province in the China Procuratorial Yearbook disclose the number of corruption crimes, crimes of bribery and malfeasance, and other crimes investigated by local procuratorial organs each year. This study used this data to characterize the degree of corruption in various regions. To eliminate the influence of the scale of the government, this study finally used the number of duty crime cases per 10,000 public servants as the proxy variable of corruption (COR), which takes the form of a natural logarithm.

(4) Other variables that affect environmental regulations. This study selected the following control variables: economic development level (Y), represented by the natural logarithm of real GDP per capita. The existing literature shows that the level of regional economic development is an important influencing factor of environmental regulation [59]. With the growth of regional economy, residents’ demand for environmental quality gradually increases, and the industrial structure of the region is more optimized, which leads to the rise of regional environmental standards and the strengthening of actual environmental regulation. Therefore, this paper introduces the level of economic development into the model.

The industrial structure (IS) may also influence environmental regulation. The regional industrial structure reflects the regional output and employment structure, so local governments must take into account the impact of local output and employment when formulating environmental regulation policies. Therefore, it is necessary to introduce the industrial structure into the explanatory variables for research concentrated on China, which is a developing country and has a central focus on economic development [60,61]. We measured the industrial structure using the proportion of the added value of secondary industry in GDP.

Previous studies have shown that population size is one of the important factors affecting the formulation of regional environmental policies [61,62]. Due to the huge differences in land area and population size among different Chinese provinces, we used the population density (PD), which is represented by the logarithm of the population at the end of the year per unit of land area, to represent the impact of population size on environmental regulation.

The unemployment rate (UNE) may also affect environmental policy, especially in areas with high concentrations of polluting industries. In these areas, high unemployment rate may force governments to relax environmental regulations to ensure production and employment in high-polluting industries [63,64]. We measured the unemployment rate using the registered unemployment rate in cities and towns.

The effect of foreign direct investment (FDI) on environmental pollution is ambiguous [59,65]. On the one hand, the increase in FDI leads to increased competition among enterprises, which in turn prompts the government to improve environmental regulations to improve social welfare (welfare effect). On the other hand, it may also lead polluting enterprises to increase bribery and reduce the level of environmental regulation (bribery effect). We measured the foreign direct investment using the proportion of foreign capital actually utilized by provinces in GDP.

### 3.3. Data Source and Description

Due to the availability limitation of Gini coefficient data, the scope of this study was limited to 26 provinces in China from 1995 to 2017 (excluding Jilin, Shandong, Hainan, and Tibet, and the data of Chongqing and Sichuan are combined). When calculating FDI data, the exchange rate (annual average price) of RMB to USD in each year was used to convert the actual use of foreign direct investment in various regions denominated in USD into RMB prices. To eliminate the influence of price factors, the data of all value forms were adjusted to the actual value under the prices of 1995, and all absolute value data were taken as natural logarithms to eliminate the influence of different dimensions. Relevant data was taken from China Environmental Yearbook, China Environmental Statistics Yearbook, China Statistical Yearbook, China Regional Economic Statistics Yearbook, China Labor Statistics Yearbook, China Procuratorial Yearbook, and provincial statistical yearbooks. The descriptive statistics of each variable are shown in Table 1.

The treatment of the endogeneity problem is an important aspect of this paper. Specifically, two measures were taken to reduce endogeneity. Firstly, we added the regional dummy variable $u_{i}$ to the regression model in Equation (3) to eliminate the impact of missing variables that are related to the region. Secondly, variables such as economic development level, industrial structure, population density, unemployment rate, and foreign direct investment that may affect environmental regulation were added to the model, which also helps to eliminate the impact of missing variables.

### 3.4. Regression Results and Analysis

Before the panel threshold regression, it should first be determined whether there is a threshold effect. If there is a threshold effect, then the number of thresholds should be determined. Taking the single threshold model in Equation (3) as an example, the following null hypothesis “$H_{0}:\beta_{11}=\beta_{12}$” can be tested for the existence of a threshold effect. If the null hypothesis is true, there is no threshold effect, otherwise there is a threshold effect. Then we can further test the threshold value, and use the likelihood ratio (LR) test statistic proposed by Hansen [43] to calculate the confidence interval of the threshold value.

In this study, we estimated the threshold effect under the null hypothesis of no threshold and a threshold, respectively, and tested the estimated threshold value. F-test statistics, critical values, and threshold values for each threshold effect are shown in Table 2.

Table 2 shows a single threshold effect at the 10% significance level when the threshold dependent variable is the Gini coefficient of the income of all residents (G) or the Gini coefficient of the income of urban residents (GU), a single threshold effect at the 5% significance level when the threshold dependent variable is the Gini coefficient of income of rural residents (GV), and a single threshold effect at the 1% significance level when the threshold dependent variable is the measurement variable of the urban–rural income inequality (UV). The estimated threshold value regarding the four threshold dependent variables G, GU, GV, and UV is 3.22, 3.6, 3.22, and 3.6, respectively. Table 2 also shows that no statistically significant double threshold effect exists regarding all four threshold dependent variables.

Next, we can estimate the parameters of the panel threshold model. To illustrate the parameter estimation method of Equation (3), we combine the piecewise function of Equation (3) as follows:

(8) $$ER_{it}=\beta_{0}+\beta_{11}\underset{=m_{1}}{\underbrace{Gini_{it}\cdot I(COR\leq \mu )}}+\beta_{12}\underset{=m_{2}}{\underbrace{Gini_{it}\cdot I(COR>\mu )}}+\beta_{2}COR_{it}+\beta_{3}Z_{it}+u_{i}+\varepsilon_{it}$$

In Equation (8), I() is an indicative function and takes the value 1 if the expression in the parentheses after it is true, and 0 otherwise. This is clearly a nonlinear function. However, if μ is known, Equation (8) can be converted into a linear regression model by defining $m_{1}=Gini_{it}\cdot I(COR\leq \mu )$ and $m_{2}=Gini_{it}\cdot I(COR>\mu )$:

(9) $$ER_{it}=\beta_{0}+\beta_{11}m_{1}+\beta_{12}m_{2}+\beta_{2}COR_{it}+\beta_{3}Z_{it}+u_{i}+\varepsilon_{it}$$

Following Hansen [43], this study used the two-step method to estimate the parameters in Equation (9). First, given μ, Equation (9) is estimated using the ordinary least square (OLS) method, and we can obtain the estimated coefficients ${\hat{\beta }}_{11}(\mu )$, ${\hat{\beta }}_{12}(\mu )$ and the concentrated sum of squared residuals SSR(μ). Second, we choose the value of $\hat{\mu }$ that can minimize $SSR(\hat{\mu })$. Finally, the estimated coefficients ${\hat{\beta }}_{11}(\hat{\mu })$ and ${\hat{\beta }}_{12}(\hat{\mu })$ are obtained.

Table 3 shows the basic regression results of environmental regulation indicators using “the proportion of industrial pollution investment in industrial added value”. Among these results, columns (1), (2), (3), and (4) are represented by the Gini coefficient of all residents’ income G, the Gini coefficient of urban residents’ income GU, the Gini coefficient of rural residents’ income GV, and the ratio of urban and rural residents’ income UV, which represents empirical results of income inequality among residents. The results in Table 2 show that there is only a single threshold for the four types of residents’ income inequality indicators. Specifically, when COR≤3.22, the estimated coefficients of G and GV are positive, and when COR>3.22, they are negative. When COR≤3.6, the estimated coefficients of GU and UV are positive, and when COR>3.6, they are negative, and the coefficients in columns (1), (2), and (4) are significant at the 1% level. This shows that the impact of residents’ income inequality on environmental regulation has a corruption threshold effect. When the level of official corruption is low, the expansion of residents’ income inequality promotes stricter environmental regulations, and when the level of official corruption is higher, the expansion of residents’ income inequality causes the decrease in environmental standards. This validates the hypothesis of the theoretical analysis and is consistent with the research by He et al. [27]. This is because the comparatively lower level of corruption means that politics is more democratic, and that the government mainly formulates and implements various policies based on the demands of the majority of citizens, rather than polluting the interests of minority groups such as business owners. When the income inequality between residents widens, the majority of low- and middle-income groups cannot enjoy the benefits of material wealth, so they will choose to fight for their rights and interests in other fields, such as urging the government to raise environmental standards to reduce the negative effects of environmental pollution and ultimately increase personal welfare. This is especially notable when the widening income inequality between residents is directly related to highly polluting production activities, which means that residents are more willing to reduce pollution emissions. The high level of corruption means a low level of democracy. The government’s rules and regulations are mainly determined by officials who may accept bribes. The collusion of government and enterprises delays the introduction of environmental protection policies and weakens the environmental regulations. When the income inequality widens, interest groups such as polluting business owners can use more funds to bribe corrupt officials to weaken environmental regulations, so pollution emissions rise.

The estimated coefficients of official corruption (COR) are all significantly negative at least at the 10% level, which indicates that official corruption not only influences the effect of residents’ income inequality on environmental regulation, but also affects environmental regulation itself. Specifically, the higher the level of official corruption, the lower the environmental standards. Among the control variables, the estimated coefficients of the economic development level (Y) are all positive, but only significant in (2) and (3). This suggests that higher levels of economic development will strengthen environmental regulation, which is consistent with research by Cole et al. [59], Apergis [66], and Cheng et al. [67]. With the improvement of the economic level, local governments and the general public pay more attention to environmental issues, and local environmental policies, technical standards, and regulatory supervision are also constantly improved, which together result in a strengthening of environmental regulation. The estimated coefficients of industrial structure (IS) are significantly negative at the level of 1%, indicating that the provinces with a higher proportion of the added value of secondary industry in GDP have weaker environmental standards, which is consistent with research by Zhang et al. [61] and Cheng et al. [67]. Because secondary industries such as textiles, paper and paper products, chemical raw materials, and chemical manufacturing are all heavily polluting industries, to pursue a higher GDP growth rate, local governments are willing to reduce environmental standards to maintain the production and operation of these highly polluting industries. The coefficients of population density (PD) are negative and significant at the level of 1%, which means that the increase in population density reduces local environmental regulation intensity and supports the conclusions of existing studies [61,62]. This is because the market demand for products in densely populated areas is large, and there are many corresponding industrial enterprises, so the environmental standards are low. The coefficients of the unemployment rate (UNE) are all negative but did not pass the significance test. It is generally believed that when the unemployment rate is high, the local government often temporarily ignores environmental issues to ensure employment priority and promote growth [63,64]; however, the empirical results of this study do not support this statement. This may be because the solution to the unemployment problem is closely related to the local economic structure, and the tertiary industry is far superior to the highly polluting industry in providing employment opportunities, so it does not necessarily result in a decline in environmental standards. The coefficients of foreign direct investment (FDI) are all positive but fail the significance test, indicating that FDI does not significantly affect environmental regulations. The existing literature on the relationship between FDI and environmental regulation mainly discusses how environmental regulation affects FDI [68,69,70,71,72,73]. The conclusion is generally that local governments are competing to reduce environmental regulation standards in order to attract foreign investment, that is, “race to the bottom” [74]. In the few studies about how FDI affects environmental regulations, Cole et al. [59] show that the effect of FDI on environmental regulation is influenced by the level of regional corruption: when the level of corruption is high, FDI reduces the intensity of environmental regulation, and vice versa. In contrast, Cole and Fredriksson [75] and Dong et al. [65] indicate that the effect of FDI on environmental regulation depends on the number of legislative units in the host country and the market sizes, respectively. The current study finds that FDI does not significantly affect environmental regulations, which is inconsistent with the literature mentioned above.

### 3.5. Robustness Test

Table 4 shows the results of the robustness test using the proportion of sewage charge income in industrial added value (ER2) as an alternative measure of environmental regulation. Similarly, columns (5), (6), (7), and (8) are the empirical results representing the income inequality of residents with G, GU, GV, and UV, respectively. Specifically, when COR≤3.75, the estimated coefficients of G and UV are positive, and negative when COR>3.75. When COR≤3.317, the estimated coefficients of GU and GV are positive, and when COR>3.317, they are negative, and the coefficients in columns (5), (6), and (8) are significant at least at the 10% level. This shows that the residents’ income inequality also has a corruption threshold effect on the environmental regulation expressed by the sewage fee income. The estimated coefficients of other control variables are basically consistent with Table 3, which shows the robustness of the empirical results.

By comparing the significance of the four income distribution gap indicators in Table 3 and Table 4, it can be seen that in addition to the rural residents’ income Gini coefficient GV, the coefficient estimates of the all residents’ income Gini coefficient G, the urban residents’ income Gini coefficient GU, and the urban and rural residents’ income ratio UV are all statistically significant. This indicates that the corruption threshold effect of all residents’ income inequality on environmental regulation is mainly generated by the urban residents’ income inequality and the urban–rural income inequality. The possible reason is that at this stage the government mainly considers the demands of urban residents and the imbalance between urban and rural economic development, whereas it does not give enough attention to the environmental protection requirements of rural residents.

## 4. Conclusions and Policy Recommendations

This paper uses panel data from 26 provinces in China dating from 1995 to 2017 to explore the impact of residents’ income inequality on environmental regulations in the presence of official corruption. We find that when the level of corruption of officials is low, the expansion of residents’ income inequality promotes stricter environmental regulations, whereas when the problem of corruption is more serious, the expansion of residents’ income inequality leads to the decline in environmental standards; that is, there is a threshold effect of corruption of the impact of residents’ income inequality on environmental regulations. This validates the hypothesis of the theoretical analysis. Further, the corruption threshold effect of all residents’ income inequality on environmental regulation is mainly generated by the urban residents’ income inequality and the urban–rural income inequality. This paper contributes to the literature that concentrates on the relationship between income inequality and environmental regulation, and shows that corruption is a key factor that can deeply influence that relationship.

This paper may provide some guidance for future research in this field. Specifically, the research is limited to Chinese provinces, and the conclusions can only offer lessons for emerging countries such as China, rather than developed or undeveloped countries. In addition, China’s unique political system also limits the application of the research conclusions to other countries. Therefore, future studies could use cross-country data to reach more general conclusions.

According to the Corruption Perception Index compiled each year by Transparency International, China has been ranked about 80th over the past five years, and has been seen to suffer from a significant corruption problem. Combined with the previous introduction to the income inequality of residents, China is currently in the situation of having high levels of both income inequality and corruption. In China’s current situation, the environmental regulations can only be strengthened by reducing the level of corruption. This is because, according to the research results of this study, when the level of official corruption is low, high income inequality can strengthen the environmental regulations.

## Figures and Tables

**Table 1:** Descriptive statistics of variables.

Variable	Unit	Average	Standard Deviation	Min	Max
*ER1*	%	0.4963	0.3843	0.0359	2.7262
*ER2*	%	0.1645	0.1089	0.0102	0.8812
*COR*	Cases/10,000 public servants	3.3553	0.3539	2.1198	4.9345
*G*	-	0.3764	0.0589	0.2275	0.4907
*GU*	-	0.2725	0.0412	0.1352	0.389
*GV*	-	0.3054	0.0469	0.154	0.4147
*UV*	-	2.8954	0.6562	1.5991	4.7585
*Y*	Yuan/person	9.2562	0.7682	7.5245	11.3177
*IS*	%	38.3084	7.8895	13.4656	52.9793
*PD*	People/km^2^	5.3447	1.3156	1.9027	8.2304
*UNE*	%	3.453	0.8866	0.46	6.8
*FDI*	%	3.107	3.0902	0.0682	16.4625

**Table 2:** Results of threshold effect tests and threshold estimators.

Threshold Independent Variable	Threshold Dependent Variable	Threshold Effect	F-Statistics	Critical Values			Threshold Value	95% Confidence Interval
				1%	5%	10%		
*COR*	*G*	Single threshold	7.38 *	18.44	9.09	6.70	3.22	(2.49, 4.05)
		Double threshold	3.14	9.08	5.01	3.69	3.38	(2.91, 3.60)
	*GU*	Single threshold	7.09 *	15.23	8.98	6.99	3.60	(2.95, 3.89)
		Double threshold	5.20	14.66	7.67	5.53	3.67	(3.20, 3.77)
	*GV*	Single threshold	13.23 **	16.67	12.54	8.99	3.22	(3.14, 3.37)
		Double threshold	4.36	14.82	9.83	6.56	3.07	(2.81, 3.17)
	*UV*	Single threshold	14.82 ***	14.07	8.08	4.99	3.60	(3.01, 4.17)
		Double threshold	3.55	13.09	8.72	6.09	3.65	(3.66, 3.83)

Note: 300 bootstrap replications are employed for each of the bootstrap tests. ***, **, and * indicate significant at 1%, 5%, and 10% levels, respectively.

**Table 3:** Baseline regression results.

Variable	(1)	(2)	(3)	(4)
*G* (COR≤3.22)	1.631 *** (0.5782)			
*G* (COR>3.22)	−1.8908 *** (0.5694)			
*GU* (COR≤3.6)		1.5655 *** (0.527)		
*GU* (COR>3.6)		−1.9326 *** (0.5406)		
*GV* (COR≤3.22)			0.2626 (0.4439)	
*GV* (COR>3.22)			−0.6385 (0.4501)	
*UV* (COR≤3.6)				0.2532 *** (0.0645)
*UV* (COR>3.6)				−0.2171 *** (0.0623)
*COR*	−0.0378 ** (0.0748)	−0.0126 ** (0.0802)	−0.0302 ** (0.077)	−0.0908 * (0.0666)
*Y*	0.0995 (0.0846)	0.1407 * (0.0825)	0.1676 ** (0.0821)	0.096 (0.0832)
*IS*	−0.0192 *** (0.0031)	−0.0189 *** (0.0031)	−0.0198 *** (0.0031)	−0.0217 *** (0.0031)
*PD*	−0.9442 *** (0.271)	−1.1253 *** (0.2679)	−0.9024 *** (0.2758)	−1.0536 *** (0.2615)
*UNE*	−0.002 (0.0272)	−0.0139 (0.0256)	−0.0274 (0.0256)	−0.0151 (0.0256)
*FDI*	0.0055 (0.011)	0.0089 (0.0099)	0.0045 (0.0113)	0.0081 (0.0099)

Note: Robust standard deviations are in parentheses. ***, **, and * indicate significant at 1%, 5%, and 10% levels, respectively.

**Table 4:** Robustness test—alternative indicator of environmental regulation.

Variable	(5)	(6)	(7)	(8)
*G* (COR≤3.75)	0.5303 ***(0.1563)			
*G* (COR>3.75)	−0.4233 *** (0.1645)			
*GU* (COR≤3.317)		0.4011 *** (0.1443)		
*GU* (COR>3.317)		−0.2579 * (0.1564)		
*GV* (COR≤3.317)			0.1763 (0.1225)	
*GV* (COR>3.317)			−0.0624 (0.1273)	
*UV* (COR≤3.75)				0.0626 *** (0.017)
*UV* (COR>3.75)				−0.0464 ** (0.0189)
*COR*	−0.0548 *** (0.0207)	−0.0628 *** (0.0203)	−0.0611 *** (0.0207)	−0.0544 *** (0.0204)
*Y*	0.001 (0.0233)	0.0081 (0.0227)	0.0196 (0.0227)	0.0021 (0.0227)
*IS*	−0.0038 *** (0.0008)	−0.0036 *** (0.0009)	−0.004 *** (0.0009)	−0.004 *** (0.0008)
*PD*	−0.0162 (0.0746)	−0.0524 (0.0736)	−0.0106 (0.0759)	−0.0172 (0.0715)
*UNE*	−0.0052 (0.0075)	−0.0005 (0.0071)	−0.0031 (0.007)	−0.0017 (0.007)
*FDI*	0.009 *** (0.003)	0.0076 *** (0.0028)	0.0091 *** (0.0031)	0.0074 *** (0.0028)

Note: Robust standard deviations are in parentheses. ***, **, and * indicate significant at 1%, 5%, and 10% levels, respectively.

## Data Availability

The data presented in this study are available on request from the corresponding author.
